# Mechanical Characterization of Human Trabecular and Formed Granulate Bone Cylinders Processed by High Hydrostatic Pressure

**DOI:** 10.3390/ma14051069

**Published:** 2021-02-25

**Authors:** Janine Waletzko-Hellwig, Michael Saemann, Marko Schulze, Bernhard Frerich, Rainer Bader, Michael Dau

**Affiliations:** 1 Department of Oral, Maxillofacial and Plastic Surgery, Rostock University Medical Center, 18057 Rostock, Germany; bernhard.frerich@med.uni-rostock.de (B.F.); michael.dau@med.uni-rostock.de (M.D.); 2 Biomechanics and Implant Technology Research Laboratory, Department of Orthopaedics, Rostock University Medical Center, 18057 Rostock, Germany; michael.saemann@med.uni-rostock.de (M.S.); rainer.bader@med.uni-rostock.de (R.B.); 3 Department of Anatomy Rostock University Medical Center, 18057 Rostock, Germany; marko.schulze@uni-bielefeld.de

**Keywords:** high hydrostatic pressure, mechanical characterization, uniaxial compression test, bone substitutes, allograft, bone regeneration

## Abstract

One main disadvantage of commercially available allogenic bone substitute materials is the altered mechanical behavior due to applied material processing, including sterilization methods like thermal processing or gamma irradiation. The use of high hydrostatic pressure (HHP) might be a gentle alternative to avoid mechanical alteration. Therefore, we compressed ground trabecular human bone to granules and, afterwards, treated them with 250 and 300 MPa for 20 and 30 min respectively. We characterized the formed bone granule cylinders (BGC) with respect to their biomechanical properties by evaluating stiffness and stress at 15% strain. Furthermore, the stiffness and yield strength of HHP-treated and native human trabecular bone cylinders (TBC) as control were evaluated. The mechanical properties of native vs. HHP-treated TBCs as well as HHP-treated vs. untreated BGCs did not differ, independent of the applied HHP magnitude and duration. Our study suggests HHP treatment as a suitable alternative to current processing techniques for allogenic bone substitutes since no negative effects on mechanical properties occurred.

## 1. Introduction

The reconstruction of severe bone defects, which originate, e.g., from infections, pathologic fractures, tumors or trauma, still remains a clinical challenge [1]. Although there are a number of different possibilities for reconstructing bone, including xenografts like demineralized bone matrices, autologous bone is still considered to be the gold standard [2,3,4]. For reconstruction surgery, autologous bone can be obtained from various donor sites such as the iliac crest, and it is specified as osteogenic, osteoinductive, osteoconductive and biocompatible, with low immunological potential and adequate mechanical strength [5]. Most other bone substitutes cannot comply with all of these requirements. Nevertheless, harvesting autologous bone is naturally limited, the occurrence of donor-side morbidities is not unusual and it requires an advanced surgical procedure [2]. The most frequently chosen alternatives to autografts are allografts [5]. Allogenic bone grafts have osteoconductive properties and avoid donor-side morbidity in the recipient. Additionally, customized types of allografts like blocks, stripes or granules are possible [2]. However, postoperative infections due to residual microbiota, proteins, etc. following allograft transplantation of human origin are a risk [6]. To prevent this, different sterilization methods including thermal processing, gamma radiation or physical and chemical decellularization have been established in recent years. Due to the removal of cellular components, any osteogenic properties are lost. However, this circumstance can be overcome with the revitalization of the graft using the recipient’s own stem cells [6,7]. Unfortunately, the mechanical strength of the allografts usually suffers when using common decellularization and sterilization methods [6].

A reasonable alternative to current decellularization methods could be treatment with high hydrostatic pressure (HHP). HHP is commonly associated with processing of food and beverages [8]. This process inactivates microbes by membrane modifications, deactivation of key enzymes and inhibition of relevant metabolic processes like protein biosynthesis [9]. In comparison to conventional thermal food processing, HHP has the advantage that flavors and vitamins are unaffected by pressures up to 800 MPa [9,10]. In recent years, HHP has gained attention in pharmaceutical research. Rigaldie et al. have shown that HHP can be used to sterilize sensitive drugs like insulin with no effect on molecular integrity [11]. Furthermore, it was shown that HHP had a devitalizing effect on different mammalian cell lines, ex situ and in situ [12,13]. In the latter, it was already shown that this form of devitalization had no negative influence on the mechanical behavior of e.g., blood vessels [13].

The aim of our present study was to evaluate the mechanical properties of human trabecular bone cylinders (TBC) and bone granules pressed to cylinders (bone granule cylinders, BGC), both treated with HHP. While TBCs with an interconnected, trabecular structure can be used for larger bone defects, the use of BGCs as filling material for non-load-bearing bone defects is conceivable. A previous study at the cellular level showed that osteoblasts, as part of trabecular bone, follow either apoptotic or necrotic means of cell death, depending on the applied HHP magnitude. A pressure range of 100–150 MPa for 10 min did not have a negative influence on the metabolic activity and cell death could not be detected. Applied pressures of 250 MPa and more led to a significant reduction in metabolic activity compared to the control group. However, it was found that a pressure of 250–300 MPa tended to lead to apoptosis, while a pressure of 450–500 MPa had a necrotic effect on the osteoblasts [14]. The level and duration of HHP applied to tissues should be selected carefully, as necrosis can be a crucial factor in clinical transplantation due to the conceivably strong immunological response of the recipient [12]. Relying on the previous cell-based study, pressures of 250 and 300 MPa were used in the present experiments, as it was assumed that the biological effects would be similar. However, due to the changes in sample geometry compared to the cell pellets, the treatment periods for TBCs and BGCs were increased from 10 min to 20 and 30 min, respectively. The mechanical properties were analyzed by performing uniaxial compression tests and comparing stiffness and strength.

## 2. Materials and Methods

### 2.1. Sample Preparation, HHP Treatment and Creation Granules-Based Bone Cylinders

Trabecular bone specimens were taken post-mortem from human femur condyles and the femoral heads of body donors (Institute of Anatomy, Rostock University Medical Center; ethics approval A 2016-0083). Both were harvested within 72 h post-mortem in order to prevent the samples from being affected by decomposition processes. Afterward, all samples were rinsed once with sterile phosphate-buffered saline (PBS) (Sigma Aldrich, Munich, Germany), supplemented with 1% penicillin/streptomycin (Sigma Aldrich, Munich, Germany). Femur condyles and femoral heads were stored at −20 °C and covered with cling film until further preparation for HHP treatment and mechanical testing.

**Figure 1:**
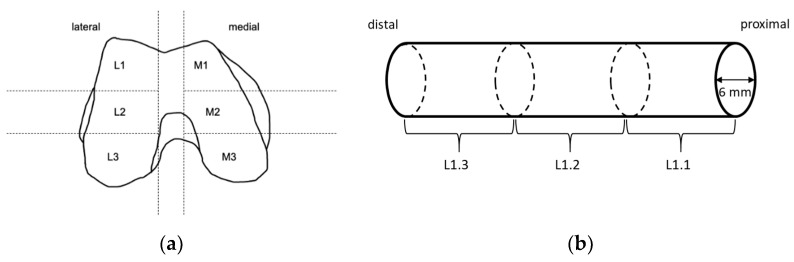
Sample preparation: (**a**) Knee condyles of human femurs were partitioned into the shown sections. Within shown sections, long cylinders were drilled along the femoral axis from proximal to distal using a trepan drill. (**b**) Long cylinders were sectioned into smaller ones with a length of 10 mm and a diameter of 6 mm using a scalpel, and care was taken that the ends were parallel to each other with a deviation of less than 5°.

Before preparation of the TBCs for the compression tests, femoral condyles were slowly thawed at 4 °C. The defrosted condyles were partitioned into different sections (Figure 1). Within each predefined section, cylinders with a diameter of 6 mm were obtained from the proximal side using a trepan drill (Ustomed Instrumente, Tuttlingen, Germany). This was performed at room temperature under constant cooling with physiological saline solution (B. Braun, Melsungen, Germany) to prevent damage from heat. The plane ends of the cylinders were rectified with the help of a scalpel to achieve parallel end faces perpendicular to the drilling axis and to shorten cylinders to a length of approximately 10 mm. Care was taken to ensure that specimens consisted of only trabecular bone, and that the ends of the cylinders were parallel to each other with a deviation of less than 5°. Specimens that did not satisfy these criteria were discarded.

For the compression test, trabecular cylinders from the identical harvesting location underwent different HHP treatments (control: *n* = 20; group A: 250 MPa, 20 min, *n* = 18; group B: 250 MPa, 30 min, *n* = 19; group C: 300 MPa, 20 min, *n* = 16; group D: 300 MPa, 30 min, *n* = 14) and were tested afterwards and compared one by one. Therefore, if a cylinder from the lateral region of a left femur condyle was taken for HHP treatment, the corresponding cylinder from the lateral region of a right femur was used as a control. The different group sizes arose due to the rejection of samples that did not satisfy the above-mentioned criteria and the alternating approach described before.

To investigate the influence of HHP on the mechanical properties of the bone granule cylinders (BGCs), the femoral heads were sawed into bone blocks with a size ranging between 0.05 and 0.1 cm^3^. Afterward, the bone blocks were processed by a bone mill (Ustomed Instrumente, Tuttlingen, Germany) to granules with a size between 1 and 2 mm.

For HHP treatment, the granules were transferred into 2 mL cryogenic tubes filled with sterile PBS. Different treatment protocols (control: *n* = 7; group A: 250 MPa, 20 min, *n* = 9; group B: 250 MPa, 30 min, *n* = 10; group C: 300 MPa, 20 min, *n* = 8; group D: 300 MPa, 30 min, *n* = 6) were applied at a constant temperature of 30 °C. The untreated specimens of the control group were stored for the same time in PBS at 30 °C.

**Figure 2:**
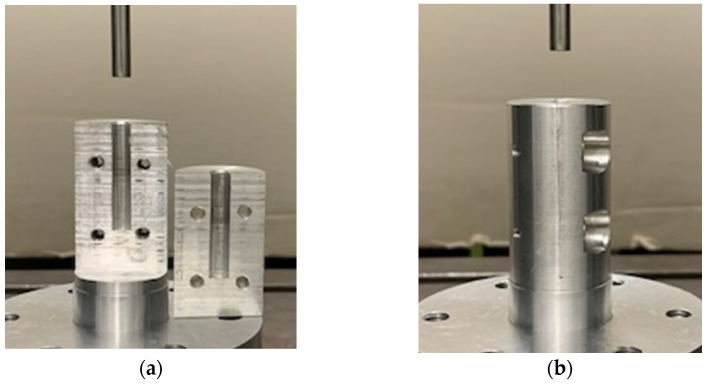
(**a**) Two segments of the hollow cylindrical body with a diameter of 6 mm. (**b**) Both segments screwed together to form a hollow cylinder. Bone granules were placed into the hollow cylinder and pressed together with a testing machine.

**Figure 3:**
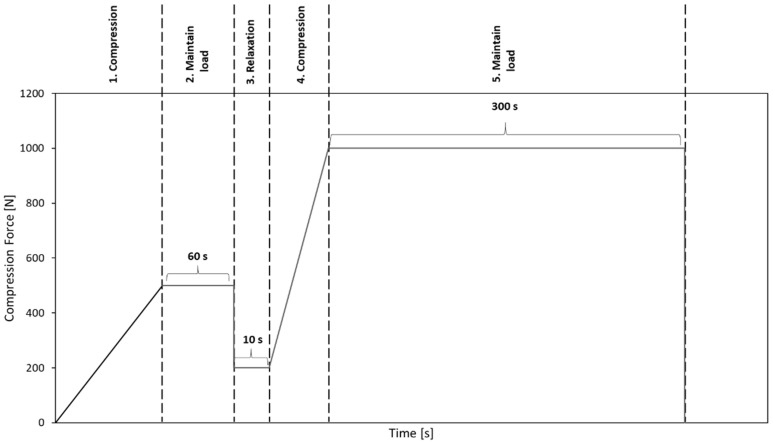
Predefined compression regime to compact bone granules to cylinders.

Before performing the uniaxial compression test, the bone granules were pressed to cylinders with a diameter of 6 mm and a length of about 10 to 12 mm. To generate cylinders of similar density, between 0.75 and 1 g of granules per cylinder were put into a hollow cylinder (Figure 2) and compressed with a uniaxial testing machine (ZwickRoell, Ulm, Germany) using a predefined compression regime (Figure 3). A compression speed of 0.5 mm/s was applied, and the compression stopped after reaching an end load of 1000 N for 5 min. Afterwards, the cylinders formed from the granules were taken out of the hollow cylinders and stored at room temperature until performing the unconfined uniaxial compression test.

### 2.2. Unconfined Uniaxial Compression Test

**Figure 4:**
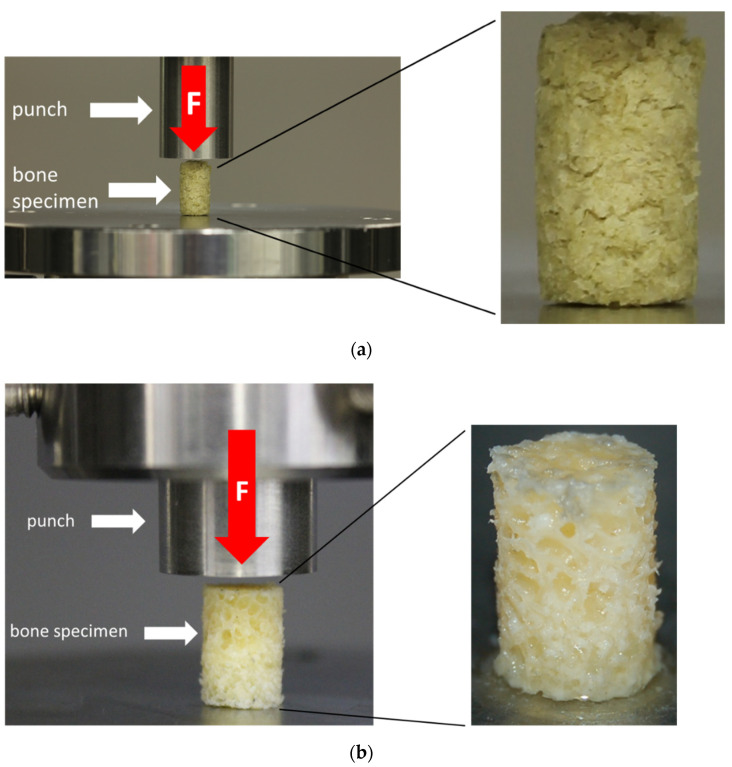
Test setup for the uniaxial unconfined compression test for the bone granule cylinders (BGCs) (**a**) and trabecular bone cylinders (TBCs) (**b**) with a representation of the respective test specimens.

The unconfined uniaxial compression tests were conducted at room temperature using a uniaxial testing machine (Z050, ZwickRoell, Ulm, Germany) and a 2.5 kN load cell (ZwickRoell, Ulm, Germany). A preload of 0.1 N was applied at a test speed of 0.05 mm/s, which was chosen based on previous studies, and which represents a physiological range [15,16]. The test runs were terminated at an engineering strain of 80%. The test setup is shown in Figure 4. TBCs and BGCs that lost their axial alignment during the uniaxial compression test were discarded.

### 2.3. Evaluation of the Results and Statistics

For the TBCs, the stiffness and the yield strength (first stress maximum after linear behavior) were compared. For BGCs, the stiffness and the stress at 15% strain were compared because the stress–strain curves of the bone granule specimens did not exhibit local maxima due to the lack of an interconnected trabecular structure.

For all human bone specimens, the generated engineering stress–strain curves were analyzed using a self-developed MATLAB script (v. R2018a, MathWorks, Natick, MA, USA). The linear-elastic region of the stress-strain curves was automatically identified and used for calculation of the stiffness via regression. For TBCs, the yield strength was identified as the first local dominant maximum after linear behavior. For BGCs, the engineering stress at 15% strain was calculated as a comparable alternative to the yield strength. Additionally, all curves of each group were averaged using Origin (v. 2018b, OriginLab, Northampton, MA, USA).

Statistical analyses were done by one-way ANOVA tests using GraphPad Prism Version 7 (GraphPad Software, San Diego, CA, USA), and results are presented as box-and-whisker plots. *p*-values ≤ 0.05 were seen as significant.

## 3. Results

### 3.1. Effects of HHP Treatment on the Mechanical Properties of Trabecular Bone Cylinders (TBCs)

**Figure 5:**
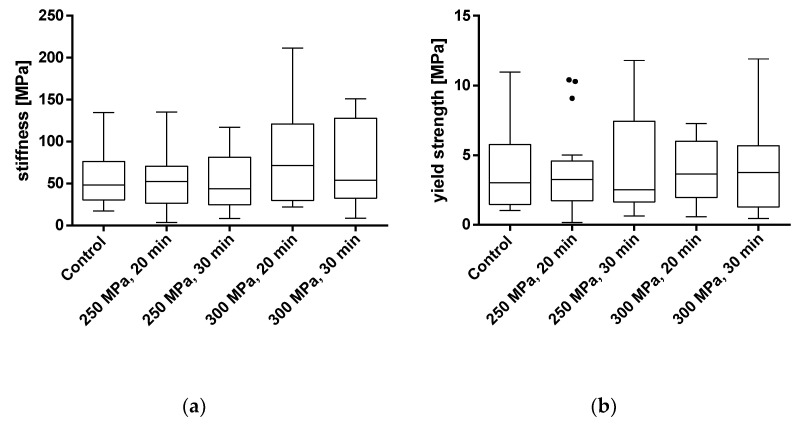
Analysis of stiffness (**a**) and yield strength (**b**) of trabecular bone cylinders (TBC) treated with and without high hydrostatic pressure (HHP). Mechanical properties were tested using a uniaxial compression test. Data are shown as box plots with median and interquartile ranges from 25 to 75%. Statistical analyses were performed using a one-way ANOVA. Sample size: control group (*n* = 20); 250 MPa, 20 min (*n* = 18); 250 MPa, 30 min (*n* = 19); 300 MPa, 20 min (*n* = 16); 300 MPa, 30 min (*n* = 14).

**Figure 6:**
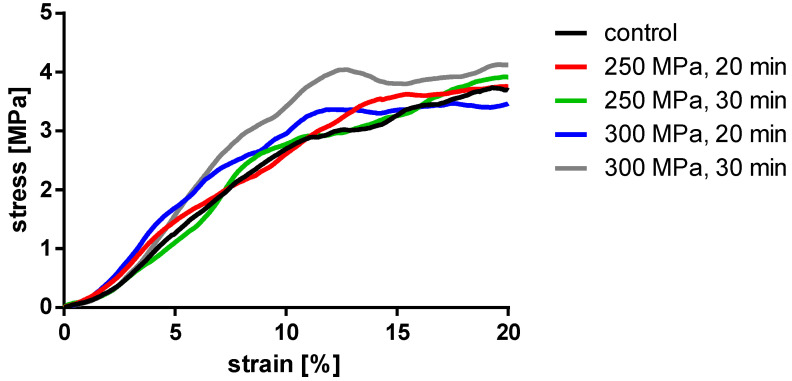
Averaged stress-strain curves of the compressed TBCs.

To evaluate the effects of HHP treatment on the mechanical properties of TBCs, samples were treated with HHPs of 250 and 300 MPa for 20 and 30 min each. The parameters of stiffness and yield strength were used for mechanical characterization. Results from samples that were tilted and/or slipped during testing or showed macroscopic defects were excluded. All results are shown as box plots in Figure 5 and summarized in Table 1. Additionally, a summary of the averaged stress-strain curves for all tested groups is shown in Figure 6.

Analyses showed no significant differences between the untreated and HHP-treated specimens (TBCs and BGCs), neither for stiffness nor yield strength. Comparing the different HHP magnitudes and durations applied to the specimens, no significant differences were determined within the treated groups. Considering the curves averaged within each group in Figure 6, it is shown that the courses of the stress–strain curves are similar. No effects of the HHP treatments can be observed in the stress–strain curves.

### 3.2. Compression of Granules to Cylindrical Samples

**Figure 7:**
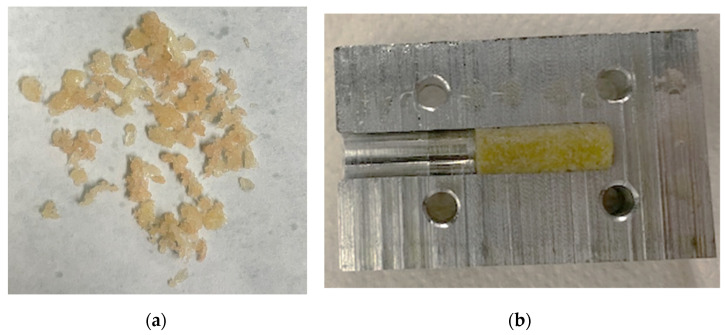
Bone granules with an average size of 1 to 2 mm (**a**). These were compressed to cylindrical samples using a hollow cylinder (**b**).

Pressed granulate bone cylinders were prepared using the described setup and technique and resulted in a length between 8 and 12 mm. An example of the compressed granules can be found in Figure 7.

### 3.3. Effect of HHP Treatment on the Mechanical Properties of Granules Bone Cylinders

**Figure 8:**
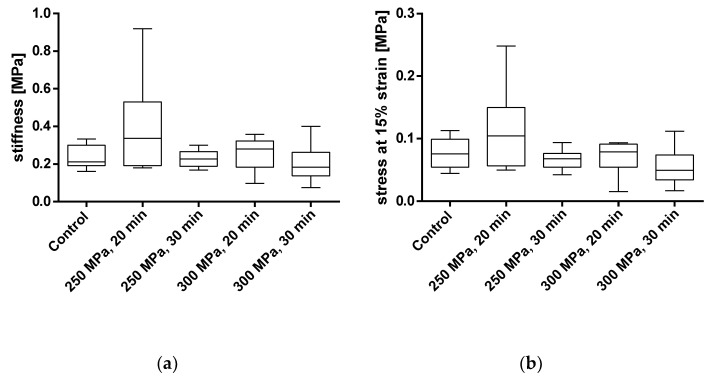
Analysis of stiffness (**a**) and stress at 15% strain (**b**) of pressed bone granules treated with and without HHP. Mechanical properties were tested using a uniaxial compression test. Data are shown as box plots with median and interquartile ranges from 25 to 75%. Statistical analyses were performed using a one-way ANOVA. Sample sizes: control group (*n* = 7); 250 MPa, 20 min (*n* = 10); 250 MPa, 30 min (*n* = 10); 300 MPa, 20 min (*n* = 8); 300 MPa, 30 min (*n* = 6).

**Figure 9:**
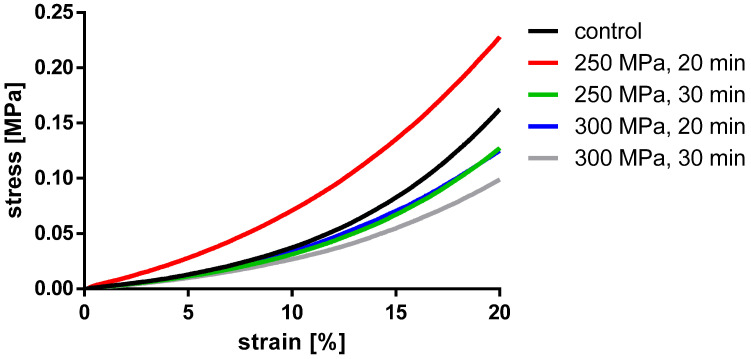
Averaged engineering stress-strain curves of pressed BGCs.

To assess the influence of HHP on the mechanical properties of the BGCs, stiffness and stress at 15% strain were chosen as comparative parameters. The results are shown in Figure 8 and in Table 2. The averaged stress–strain curves for granulated bone cylinders are shown in Figure 9.

For BGCs, neither the stiffness nor the strain at 15% stress showed any significant differences between the groups. Figure 9 shows, as for the TBCs, the averaged stress-strain curves for granulate bone cylinders. Here, too, a similar course for all groups can be determined.

Comparing the stiffness of TBCs and BGCs, the latter comprises only a fraction of the native cylinders due to its missing intertrabecular structure.

## 4. Discussion

The reconstruction of bone defects is still challenging. In particular, cases that are correlated with severe bone loss or cases of patients with disorders in healing processes are clinically demanding [5]. Autologous bone is still considered to be the gold standard for bone defect reconstruction despite the known donor site morbidity and the limited amount of harvestable autologous bone [17]. A major drawback of the alternative, allogenic bone, which is less limited in quantity, is the alteration of mechanical properties due to current devitalization and sterilization methods, including thermal processing and gamma irradiation [18,19,20].

HHP as a gentle devitalization and sterilization method has been used in the food industry for several years, but it has also gained attention in the medical and pharmaceutical sectors [9]. Depending on the applied pressure, various studies have shown that mammalian cells can be devitalized while preserving an intact tissue matrix, which has already been shown for blood vessels and uterine tissues [12,13,21]. HHP has the potential to serve as a novel way to process allogenic bone substitute materials.

Within this study, it was shown that HHP had no effect on the macroscopic mechanical properties of human trabecular bone, as had already been shown for other tissues [13,21,22]. Specifically, HHP-treated TBCs showed no significant differences in either stiffness or yield strength. It was noticeable that all groups showed high variance in their mechanical properties, which is typical for biological samples, as gender and physical conditions of tissue donors can influence the results. Nevertheless, when looking at the averaged stress–strain curves of the individual groups with very similar curve progressions, it was shown that the eventual effects of HHP were small compared to the naturally occurring effects. The range for the compressive strength of trabecular bone is specified as 2 to 48 MPa according to the literature [7]. The measured strength of TBCs in this study was at the lower end of this range, at around 4 MPa for all groups. For this reason, the effect of HHP on trabecular bone specimens from other regions that have typically denser bone than the femoral condyles should be analyzed in further studies.

For pressed BGCs, no significant differences between the groups were found with stiffness and stress at 15% strain, i.e., no changes in the mechanical properties when comparing untreated and HHP-treated groups could be observed, but they were clearly below the values of the TBCs. The stress-strain curves of the BGCs also differ substantially from that of the TBCs. These curves exhibit a continuous, monotonic progression without local maxima due to the lack of an interconnected trabecular structure. Furthermore, this results in the significantly lower strength of BGCs when compared to native trabecular specimens. In addition, with these BGCs as well as the TBCs, it is noticeable that the values for both stiffness and stress at 15% strain vary widely. Many providers of bone substitute materials advertise both granules and bone blocks, which have been processed thermally or with gamma radiation [23,24]. According to various studies, these sterilization methods greatly reduce the mechanical properties of the allografts [19]. Commonly used irradiation doses between 20 and 30 kGy do not reduce the stiffness of bone, but they significantly reduce the ultimate stress and, to a smaller extent, the bending strength [18,25]. Thermal sterilization up to 60 °C has no effect on the mechanical properties, but higher temperatures (up to 100 °C) reduce the mechanical strength significantly [19].

There are several indications as to why HHP does not seem to affect the mechanical properties of treated biological tissues, which could explain the results observed in this study. An important role in the toughness of bone is played by collagen type I, which makes up the main part of extracellular matrix proteins that could be found in trabecular bone [26]. Pivotal to the mechanical properties of bone tissue is the formation of calcium-apatite crystals in the collagen fibrils interface [27,28]. This can also be seen in the correlation between calcification and bone stiffness [29]. Diehl et al. evaluated the effect of HHP on the biological properties of extracellular matrix (ECM) proteins and showed that HHP treatment had no influence on collagen type I and other common ECM proteins, such as fibronectin and vitronectin, in regards to their biological behavior in when compared to untreated ECM proteins [30].

These findings are supported by various studies at the molecular level [31,32]. Proteins are a complex organization of subunits with primary, secondary, tertiary and quaternary structures. The successive hierarchy describes increasing complexity of organization, which is affected by HHP treatment in different ways [31,32]. The primary structure (polypeptide chain) consists of covalent bonds, which are not affected by HHP. The secondary structure of proteins, created by the formation of hydrogen bonds between the polypeptides, is irreversibly degraded at pressures higher than 700 MPa. Tertiary structures, made up of hydrophobic interactions and ionic bonds, are broken up at HHPs higher than 200 MPa, and quaternary structures, with non-covalent bonds including Van der Waal’s forces, are dissolved at HHPs between 100 and 150 MPa [31,32]. In contrast to the complete protein destruction that occurs at very high or low temperatures or during gamma irradiation, these structural changes caused by HHP are reversible at pressures between 100 and 300 MPa. This means proteins can rearrange after HHP treatment [18]. Based on this observation, some literature also describes a new association of previously incorrectly folded protein structures after HHP treatment [33,34,35].

In the present study, HHP between 250 and 300 MPa was applied, which induced reversible changes in protein structure. This could be the reason for the maintained mechanical properties of the specimens. However, our study is limited by several factors. As mentioned above, only TBCs from femoral condyles were studied. The same applies to BGCs, which were solely extracted from femoral heads. Furthermore, only one mechanical test was performed, and the specimen size did not reflect the influence of HHP on an entire femur. Additionally, only trabecular bone was analyzed in the study at hand, and the effect of HHP on cortical bone tissue should be analyzed in further studies. Another limitation is due to the use of biological samples; different tissue donors vary in physiological characteristics such as age, physical activity or pre-existing diseases. The structure and morphology of bone varies as well, which is reflected in the mechanical properties of whole bones and also bone tissue. A further limitation is contamination with bacteria and germs. The sterilization effect of HHD on these organisms was not the subject of this study.

The results presented fit well with the investigations described above. Further mechanical tests, such as a three-point-bending test, could be performed in addition to the uniaxial compression test already shown here. Samples composed of both cortical and trabecular bone tissue or whole bones could also be analyzed with regard to the effects of HHP on their mechanical properties. This could give a good overall view of the influence of HHP on bone at the macroscopic level. Simultaneously, the effects of HHP at the microscopic and molecular levels should not be neglected. Here, a structural analysis of proteins after HHP treatment and an analysis of the inorganic bone components via electron microscopy is conceivable. If HHP is discussed as an alternative to the previous methods of processing allografts, the inherent mechanical properties are of great importance. With regard to clinical applications, the devitalizing efficiency and immunological safety should also be studied in the future. In further studies, it will still be necessary to assess the effects of HHP from a microbiological and virological point of view. Here, the study of bacterial and virological load before and after HHP treatment could be conceivable following previous works [36,37].

In the case of the BGCs, the compression method could also be optimized depending on the targeted clinical application. Loosely packed granules may well have an advantage for the ingrowth of cells, and more densely packed granules could possibly be used as a load-bearing structure. In addition, compression parameters or shapes (e.g., blocks) deviating from those shown here should be investigated.

As shown in the study, the clinical use of HHP-treated TBCs and BGCs is conceivable. Although the formed granulated bone cylinders are more fragile than native trabecular bone, granulate cylinders might be used as shaping filler material for non-load-bearing bone defects, acting as osteoinductive and osteogenic scaffolds.

In conclusion, this study showed that HHP treatment has a pivotal advantage over conventional processing methods of bone substitute materials by maintaining the mechanical properties in combination with effective cell devitalization.

## 5. Patents

A patent application with the number DE 10 2020 131 181.8 was submitted to the German Patent and Trademark Office.

## Figures and Tables

**Table 1:** Overview of the results, including sample size *n*, the mean and the standard deviation for trabecular bone cylinders after the uniaxial compression test for stiffness and yield strength.

Treatment	*n*	Stiffness [MPa]		Yield Strength [MPa]	
		Mean	Standard Deviation	Mean	Standard Deviation
control	20	58.432	±35.916	3.767	±2.676
250 MPa, 20 min	18	57.364	±35.697	3.881	±3.080
250 MPa, 30 min	19	54.691	±34.732	4.461	±3.557
300 MPa, 20 min	16	80.366	±58.505	3.775	±2.272
300 MPa, 30 min	14	75.071	±49.520	4.238	±3.184

**Table 2:** Overview of the results, including the sample size *n*, the mean and standard deviation for BGCs after the uniaxial compression test for the stiffness and stress at 15% strain.

Treatment	Group Size	Stiffness		Stress at 15% Strain	
		Mean	Standard Deviation	Mean	Standard Deviation
control	7	0.239	±0.062	0.078	±0.025
250 MPa, 20 min	9	0.381	±0.246	0.110	±0.066
250 MPa, 30 min	10	0.227	±0.044	0.067	±0.015
300 MPa, 20 min	8	0.253	±0.087	0.070	±0.026
300 MPa, 30 min	6	0.203	±0.108	0.055	±0.032

## Data Availability

The data presented in this study are available on request from the corresponding author. The data are not publicly available due to the nondisclosure agreement with the project sponsor.
